# Supplementary material for: What is the overlap between malnutrition, frailty and sarcopenia in the older population? Study protocol for cross-sectional study using UK Biobank
Source: PLoS One. 2022 Dec 6;17(12):e0278371. doi: 10.1371/journal.pone.0278371 (PMC9725160; doi:10.1371/journal.pone.0278371)
Supplement: S1 Table — (DOCX) [file pone.0278371.s001.docx]

# **Supplementary information**

**Table S1 34-cumulative deficits matched to variables from UK Biobank.**

| Data-field | variables from Biobank | 36 deficits eFI |
| --- | --- | --- |
| 6164 | Types of physical activity in last 4 weeks | activity limitation |
| category 2403 | blood, blood-forming organs and certain immune disorders | anaemia & haematinic deficiency |
| 41270 | diagnoses ICD10 | ARTHRITIS |
| 131350 | first reported (atrial fibrillation and flutter) | atrial fibrillation |
| 41270 | diagnoses- ICD10 | cerebrovascular disease |
| 41270 | diagnoses- ICD10 | chronic kidney disease |
| 2443 | diabetes diagnosed by doctor | diabetes |
| 21053 | degree bothered by dizziness in the last 3 months | dizziness |
| 4717 | Shortness of breath walking on level ground | dyspnoea |
| 2296 | falls in the last year | falls |
| 120078 | Ever had an open sore on foot | foot problems |
| 3005 | fracture resulting from simple fall | fragility fracture |
| 2247 | hearing difficulty/problems | hearing impairment |
| 131354 | data I50 first reported (heart failure) | heart failure |
| 41270 | diagnoses- ICD10 | heart valve disease |
| 6146 | attendance/disability/mobility allowance | housebound |
| 41270 | diagnoses- ICD10 | hypertension |
| 41270 | diagnoses- ICD10 | hypotension/syncope |
| 41270 | diagnoses- ICD10 | ischaemic heart disease |
| 4282 | Maximum digits remembered correctly | memory and cognitive problems |
| 6146 | attendance/disability/mobility allowance* | mobility and transfer problems |
| 41270 | diagnoses- ICD10 | osteoporosis |
| 42031 | Source of all cause parkinsonism report | parkinsonism and tremor |
| 41270 | diagnoses- ICD10 | peptic ulcer |
| 41270 | diagnoses- ICD10 | peripheral vascular disease |
| 137 | number of treatments/medications taken | polypharmacy |
| 670 | Type of accommodation lived in | requirement for care |
| 41270 | diagnoses- ICD10 | respiratory disease |
| 131594 | Date K27 first reported (peptic ulcer, site unspecified) | skin ulcer |
| 21061 | degree bothered by trouble sleeping in the last 3 months | sleep disturbance |
| 670 | Type of accommodation lived in | social vulnerability |
| 41270 | diagnoses- ICD10 | thyroid disease |
| 41270 | diagnoses- ICD10 | urinary incontinence |
| 41270 | diagnoses- ICD10 | urinary system disease |
| 41270 | diagnoses- ICD10 | visual impairment |
| 2306 | Weight change compared with 1 year ago (unintentionally weight loss ) | weight loss and anorexia |

* same variable used before in housebound (two deficits has the same variable, thus the frailty index (FI) will be divided by total 35 rather than 36)
