# Supplementary material for: What is the overlap between malnutrition, frailty and sarcopenia in the older population? Study protocol for cross-sectional study using UK Biobank
Source: PLoS One. 2022 Dec 6;17(12):e0278371. doi: 10.1371/journal.pone.0278371 (PMC9725160; doi:10.1371/journal.pone.0278371)
Supplement: Attachment — Submitted filename: Response to Reviewers.docx [file pone.0278371.s003.docx]

| The authors would like to thank the reviewers for their insightful comments that have improved the quality of our manuscript. | |
| --- | --- |
| 1^st^ Reviewer comments | Response |
| In this submission, the authors propose a cross-sectional study to determine the prevalence and overlap between the related conditions of malnutrition, frailty, and sarcopenia. They will do so utilizing a subset of data from the UK Biobank database. Specifically, they will utilize data from adults 50 to 69 years old. A clear strength of the proposed study is the anticipated subject number of more than 380,000 subjects. In general, the paper was very well written. I do have a few concerns/suggestions for the authors. | Thank you |
| 1. One of the three phenotypic criteria for malnutrition indicates a cut-point for BMI of < 22 kg/m2 for adults older than 70. However, this data set will not contain subjects over 70. | The UK Biobank criteria stated participants recruited from age 49-69 years. However, the dataset has 73,863(14.7) aged 66-70 years and 7(0) aged 71-73. |
| 2. One of the etiologic criteria for malnutrition indicates that CRP and albumin will be used as indices of inflammation. Albumin is not a marker of inflammation, but could be an indicator of malnutrition. I can see including albumin as a marker of malnutrition, but the authors should not indicate that it is an indicator of inflammation. I’m referring specifically to the wording the in second paragraph of page 7. | Albumin is not reliable by itself, but is required to assess inflammatory response using CRP. I have deleted the reference to albumin to save confusion. |
| 3. The authors indicate that they are using two models to map frailty—the 36 deficits model and the phenotype model. Under the description of each (pages 7 and 8) they define the criteria for frailty, but it’s less clear how the subjects will be ultimately classified. To be considered frail, do subjects need to meet the criteria under both models (or just one)? | The two models will be used to identify frail participants. However, each model will be used separately then will compared the final results.  The phenotype model will classify frail if the participants have three criteria or more out of the five criteria’s. While, the 36-cumulative deficits model will categorised participants as frail if the frailty index(FI) more than 0.35  I have added a sentence to clarify  Two models will be used to define frailty and compared. Only one model will be used to consider the overlap with malnutrition and sarcopenia. |
| 4. Also, the supplementary table for the 36 deficits models is not well labeled. In the text it refers to Table S1. The table is not labeled. It is also not clear in the text that the BioBank does not include 3 of the 36 deficits. | Author added title to the table with footer and re-arrange it. First column is the variable number in the dataset UK Biobank, 2^nd^ column presenting the name of the variable, and the 3^rd^ column is the 36-cumulative deficits model. I hope it is clear. |
| 2^nd^ Reviewer comments | Response |
| The background gives a good overview of the topic. Methods/design are very well described, and the variables are clearly illustrated in table 1, 2. The statistical analyses that will be used are satisfactory presented. Discussion and study limitations are conclusive stating “using a secondary database not designed specifically to answer the research question resulting in selection bias in the sample". The authors also highlight the limitations because some of the variables for identification of malnutrition, frailty or sarcopenia will not be available or are self-reported. Language check! | Thank you  I have check the language here and changed slightly to improve readability. |
| Minor comments; Abstract: Discussion: This proposed study will help with understanding the presence (I suggest: clarify or help to understand). | Line 27-28: “This proposed study will help to understand the presence of malnutrition, frailty…etc”. The words have been changed per the suggestion. |
| Background, page 3, 2nd paragraph, first sentence “alterations to the senses” do you mean “in”? | Line 52-53: “The aging process involves changes in body functions including alterations in the senses of smell,”. The words have been changed per the suggestion. |
| 2nd paragraph line 3-6, rewrite. I suggest adding ref 19 (fig 3 a conceptual tree of nutritional disorders) along with ref 9 and focus on malnutrition i.e.: a) starvation derived undernutrition and b) cachexia – disease related malnutrition! It is important to include “disease-related malnutrition” in the text. | Line 55-61: “Malnutrition has two forms: undernutrition, which involves wasting, stunted growth (starvation related under-weight and cachexia or disease related malnutrition), vitamin or mineral deficiencies (micronutrient abnormalities), and being overweight or obese (overnutrition), which results from diet-related non-communicable diseases[9,10]. Cachexia is a another nutritional disorder which can be define as “a complex [metabolic syndrome](https://www-sciencedirect-com.manchester.idm.oclc.org/topics/nursing-and-health-professions/metabolic-syndrome-x) associated with underlying illness and characterized by loss of muscle with or without loss of fat mass”[11].  I have not included the diagram as I do not have permission from the journal to reproduce but I have described with the reference. |
| Page 7, line 2 some word (s) is missing here! | Line 136-138: “The EWGSOP recommends the use of below two standard deviations from the mean as reference values taken from healthy, young adults rather than other predictive reference populations cut-off points [47]. |
| Page 9, paragraph 4 second line – word (s) missing – will be collected? | Line 22-23: “Variables for malnutrition will be mapped according to the Global...etc” it has been changed. |
| Discussion, line 3. Here you may add a study performed in nursing home setting Faxén-Irving G, Luiking Y, Grönstedt H, Franzén E, Seiger Å, Vikström S, Wimo A, Boström AM, Cederholm T. Do Malnutrition, Sarcopenia and Frailty Overlap in Nursing-Home Residents? https://doi.org/10.14283/jfa.2020.45 J frailty Aging 2021;10(1)17-21. | Line 278-282: “A study measured the overlap between the three conditions in a nursing home, which showed that 7% of the 92 residents aged 75 and above had all three conditions, and 32% of the participants were free from malnutrition, frailty, and sarcopenia. In addition, 5% of participants were malnourished with sarcopenia in the nursing home. Whereas 10% patients had sarcopenia and were frail[57].” |
| Discussion, Line 7-8 word (s) missing! | Line 286-288: “This highlights early identification of these conditions in older adults is vital so treatment and interventions can initiated in this age group [61].” |
