# Supplementary material for: What is the overlap between malnutrition, frailty and sarcopenia in the older population? Study protocol for cross-sectional study using UK Biobank
Source: PLoS One. 2022 Dec 6;17(12):e0278371. doi: 10.1371/journal.pone.0278371 (PMC9725160; doi:10.1371/journal.pone.0278371)
Supplement: Attachment — Submitted filename: Rebuttal 10 Oct 2022.docx [file pone.0278371.s004.docx]

| The authors would like to thank the reviewers for their insightful comments that have improved the quality of our manuscript. | |
| --- | --- |
| 2^nd^ Reviewer comments | Response |
| The MS has improved! Some minor comments, most language: Page 3, line 55-58: “Malnutrition has two forms: undernutrition, which involves wasting, stunted growth (starvation related under-weight and cachexia or disease related malnutrition), vitamin or mineral deficiencies (micronutrient abnormalities), and being overweight or obese (overnutrition)”. My suggestion: There are (or exist) two forms of malnutrition……. | Done Line 55-56: “There are two forms of malnutrition undernutrition and being overweight or obese, which results from diet-related non-communicable diseases.” |
| Table 2. Variable mapped to malnutrition, frailty and sarcopenia. This table is difficult to read – it’s some kind of overlapping in the pdf-file. | Done, fixed the overlap between table 1 and table 2. |
| Page 10, Line 202-204: word is missing. “These variables will represent three phenotypic criteria and two etiologic criteria, where patients will be defined as having malnutrition if …..has one phenotypic criterion and one aetiological criterion. | Line 189-190: “These variables will represent three phenotypic criteria and two etiologic criteria, where patients will be defined as having malnutrition if they have one phenotypic criterion and one aetiological criterion.” |
| Page 13, Line 278-280: words are missing: “From the baseline assessment, details of the participants’ characteristics anthropometric measurements, participants’ medical histories, smoking status, uses of medications, dietary intake records and alcohol intake”…….. | Line 267-268: “From the baseline assessment, details of the participants’ characteristics anthropometric measurements, participants’ medical histories, smoking status, uses of medications, dietary intake records and alcohol intake will be used” |
| Discussion. Page 14, Line 288-292: A study measured the overlap between the three conditions in a nursing home, which showed that 7% of the 92 residents aged 75 and above had all three conditions, and 32% of the 290 participants were free from malnutrition, frailty, and sarcopenia. In addition, 5% of participants were malnourished with sarcopenia in the nursing home. Whereas 10% patients had sarcopenia and were frail 58]. My suggestion: In a group of 92 selected nursing home residents aged 75 and above all three conditions were present in 7%. Twenty-nine (32%) residents were neither malnourished, sarcopenic nor frail. In addition, 5% of the residents were malnourished and simultaneously sarcopenic and frailty coexisted with sarcopenia in 10% of the subjects. | This has been changed and thank you for the suggestion. Line 275-279:” In a group of 92 selected nursing home residents aged 75 and above all three conditions were present in 7%. Twenty-nine (32%) residents were neither malnourished, sarcopenic nor frail. In addition, 5% of the residents were malnourished and simultaneously sarcopenic and frailty coexisted with sarcopenia in 10% of the subjects.” |
| Line 296-298. word is missing: “This highlights …….early identification of these conditions in older adults is vital so treatment and interventions can initiated in this age group” | Line 288-289:” This highlights that early identification of these conditions in older adults is vital so treatment and interventions can be initiated in this age group” |
| 3^rd^ Review comments | Response |
| Authors reports that baseline assessment will be used for the study. Therefore, since participants aged between 40-69 were included, I do not expect to have participants older than 70 years in analyses. This point is unclear and deserved better explanation. | Line 144-145: “Participants were recruited from the NHS aged between 40-69 years. However, the database included participants aged 37 to 73 for the baseline visit.” |
| It is not clear why authors expect to include 76% of the total sample of the UK biobank in the analyses. Please develop. | The project protocol is to include all participants age 50 and above that is 76% of the sample size. Thus, any participants below 50 will not included in the study, which represent 24% of the sample size. This has been clarified.  Line 175:(76% of the total sample of participants in the UK Biobank, who were ≥50 years old) [29]. |
| Table 1 and Table 2 are unreadable in the PDF document I have received. Therefore I was unable to review the criteria of sarcopenia, frailty and malnutrition, which is unfortunate. | Sorry it overlapped. This issue have been fixed |
| I recommend authors to use the EWGSOP2 criteria for the diagnosis of sarcopenia. Please update criteria and cut-offs used for the diagnosis. | The analysis is currently being conducted, we will add this as a limitation and recommendation for future studies. This has now been mentioned in the discussion.  Line 327-335:” Lastly, the EWGSOP criteria was used to identify participants with sarcopenia, however, this has now been updated (EWGSOP2). There are two differences in the updated version, which are the use of muscle strength as a criterion rather than low muscle mass (65)and low physical activity has been excluded. (65). There have been a number of studies comparing EWGSOP1 and EWGSOP2, which showed good levels agreement (66-69). Other studies, indicate a lower prevalence of sarcopenia when using EWGSOP2(70), therefore, this study plans to use EWGSOP1 for identifying participants with sarcopenia.” |
| Could author inform if tools used for the measures of muscle mass and muscle strength were calibrated ? This is an important aspect. | Clarification has been added  Line 326-327: “Standard operating procedures were used for measurements at the baseline visits for anthropometry, however it is unknown if the equipment was calibrated.” |
